# Performance of a constructed wetland in Grand Marais, Manitoba, Canada: Removal of nutrients, pharmaceuticals, and antibiotic resistance genes from municipal wastewater

**DOI:** 10.1186/1752-153X-7-54

**Published:** 2013-03-18

**Authors:** Julie C Anderson, Jules C Carlson, Jennifer E Low, Jonathan K Challis, Charles S Wong, Charles W Knapp, Mark L Hanson

**Affiliations:** 1 Richardson College for the Environment, Department of Environmental Studies and Sciences and Department of Chemistry, The University of Winnipeg, Winnipeg, MB, R3B 2E9, Canada; 2 Department of Environment and Geography, University of Manitoba, Winnipeg, MB, R3T 2N2, Canada; 3 Department of Chemistry, University of Manitoba, Winnipeg, MB, R3T 2N2, Canada; 4 David Livingstone Centre for Sustainability, Department of Civil & Environmental Engineering, University of Strathclyde, Glasgow, Scotland, G1 1XN, UK

**Keywords:** Sewage lagoon, Wastewater, Treatment wetland, Antibiotic resistance genes, Pesticides, Pharmaceuticals

## Abstract

**Background:**

The discharge of complex mixtures of nutrients, organic micropollutants, and antibiotic resistance genes from treated municipal wastewater into freshwater systems are global concerns for human health and aquatic organisms. Antibiotic resistance genes (ARGs) are genes that have the ability to impart resistance to antibiotics and reduce the efficacy of antibiotics in the systems in which they are found. In the rural community of Grand Marais, Manitoba, Canada, wastewater is treated passively in a sewage lagoon prior to passage through a treatment wetland and subsequent release into surface waters. Using this facility as a model system for the Canadian Prairies, the two aims of this study were to assess: (a) the presence of nutrients, micropollutants (i.e., pesticides, pharmaceuticals), and ARGs in lagoon outputs, and (b) their potential removal by the treatment wetland prior to release to surface waters in 2012.

**Results:**

As expected, concentrations of nitrogen and phosphorus species were greatest in the lagoon and declined with movement through the wetland treatment system. Pharmaceutical and agricultural chemicals were detected at concentrations in the ng/L range. Concentrations of these compounds spiked downstream of the lagoon following discharge and attenuation was observed as the effluent migrated through the wetland system. Hazard quotients calculated for micropollutants of interest indicated minimal toxicological risk to aquatic biota, and results suggest that the wetland attenuated atrazine and carbamazepine significantly. There was no significant targeted removal of ARGs in the wetland and our data suggest that the bacterial population in this system may have genes imparting antibiotic resistance.

**Conclusions:**

The results of this study indicate that while the treatment wetland may effectively attenuate excess nutrients and remove some micropollutants and bacteria, it does not specifically target ARGs for removal. Additional studies would be beneficial to determine whether upgrades to extend retention time or alter plant community structure within the wetland would optimize removal of micropollutants and ARGs to fully characterize the utility of these systems on the Canadian Prairies.

## Background

The environmental fate of excess nutrients and pharmaceuticals and personal care products (PPCPs) has become an area of great interest over the past decade, particularly in aquatic ecosystems [1]. In general, PPCPs are designed to be biologically active at very low doses, and the effects of exposure to these compounds, particularly under chronic exposures, are not well understood [2,3]. Micropollutants such as PPCPs are not typically targeted for removal by wastewater treatment systems [3], so these compounds are detected in surface waters globally [4-6].

In addition, antibiotic resistance genes (ARGs) have also been detected in the environment as a result of the prevalent human and veterinary use of antibacterial and antimicrobial products [7-10], which are also not eliminated by conventional wastewater treatment plants [5,11]. Genes encoding for resistance to a variety of antibiotics have been detected in surface waters, sewage, treated wastewater, and drinking water, and are ubiquitous in aquatic environments impacted by human activity [10,12-15]. Over the past decade, focus has shifted from studying antibiotic resistance primarily in a clinical context to examining the potential environmental impacts of ARGs [12]. Concern and interest are growing in regards to the role and effects of ARGs in aquatic ecosystems since there are public and environmental health implications resulting from transport and dissemination of ARGs into water bodies [7,10,14,16,17]. Primarily, ARGs are a concern due to the potential for persistence of antibiotic resistance and future outbreaks via antibiotic-resistant pathogens [5,12]. The World Health Organization has identified antibiotic resistance as a major health concern [17] and it has been reported that diseases that were previously eradicated (e.g. tuberculosis) may soon pose a severe global risk to human health due to the prevalence of ARGs and resistant pathogens [18].

Treatment wetlands offer a potential option for cost-effective removal of PPCPs and ARGs from municipal wastewater. Wetlands can be used as a secondary or tertiary treatment step, following chemical and/or biological treatments, and rely upon natural processes in shallow water or temporarily flooded land that is able to support aquatic life [18]. These systems tend to be less resource-intensive than conventional wastewater treatment plants [5,18], and have been used successfully for treatment of municipal sewage in small communities, as well as for some industrial wastewaters [19]. While most research has focused on the use of wetlands for reduction of nutrients and biochemical oxygen demand (BOD) in water bodies receiving runoff from agricultural or urban sources [6,20], recent studies have shown that these systems might remove PPCPs as well [1,6,18,21]. Specifically, wetlands have shown potential for removal of antibiotics via sorption, uptake by plants, and partial or complete physico-chemical and/or biological degradation [5]. However, removal efficiency in wetlands is affected by a number of factors, including age of the wetland, seasonality, and presence or absence of plants [19-21]. Effects of climate and seasonality are particularly important considerations for wetlands in the Canadian Prairies [4,22] as many studies of treatment wetlands have been conducted in the southern United States (e.g. [1]) and Europe (e.g. [5,18]). These climates are quite different from Canada, and the published results may not be applicable to this geographical region as wetlands rely heavily on climatic and biological factors. To optimize these systems for removal of PPCPs and ARGs in the Canadian prairie climate, a better understanding of the numerous interacting parameters is required, as well as some sense of how current systems are functioning, if at all, in this regard.

Within the province of Manitoba, Canada, there are many small communities (populations ≤ 10,000) where full-scale conventional wastewater treatment plants are not financially or operationally feasible. It has been estimated that upwards of 350 communities in Manitoba rely on lagoons for the treatment of their waste prior to direct release into surface waters [23]. With the implementation of stricter provincial and federal guidelines around municipal wastewater release [24], alternative treatment systems, such as wetlands, need to be characterized for their efficacy at removing nutrients, PPCPs, and ARGs in a rural, prairie context. Preliminary work has been done in other communities in Manitoba to quantify the concentrations of pharmaceuticals in wastewater lagoon effluent [4], but the effectiveness of wetland treatment in this region is currently unknown. The community of Grand Marais uses one of the few operating sewage lagoon/constructed wetland treatment systems in the province and was selected as a model system for this study. The overall objectives of this study were to characterize the presence of nutrients and emerging wastewater contaminants (i.e., PPCPs and ARGs) in the Grand Marais system and to evaluate the effectiveness of treatment wetlands in removal of these contaminants. It was hypothesized that the use of a treatment wetland would enhance degradation and elimination of these target compounds, and therefore, could be an option to complement the current lagoon wastewater treatment system in communities that rely on lagoon treatment alone.

## Results

### General water quality parameters

**Figure 1:**
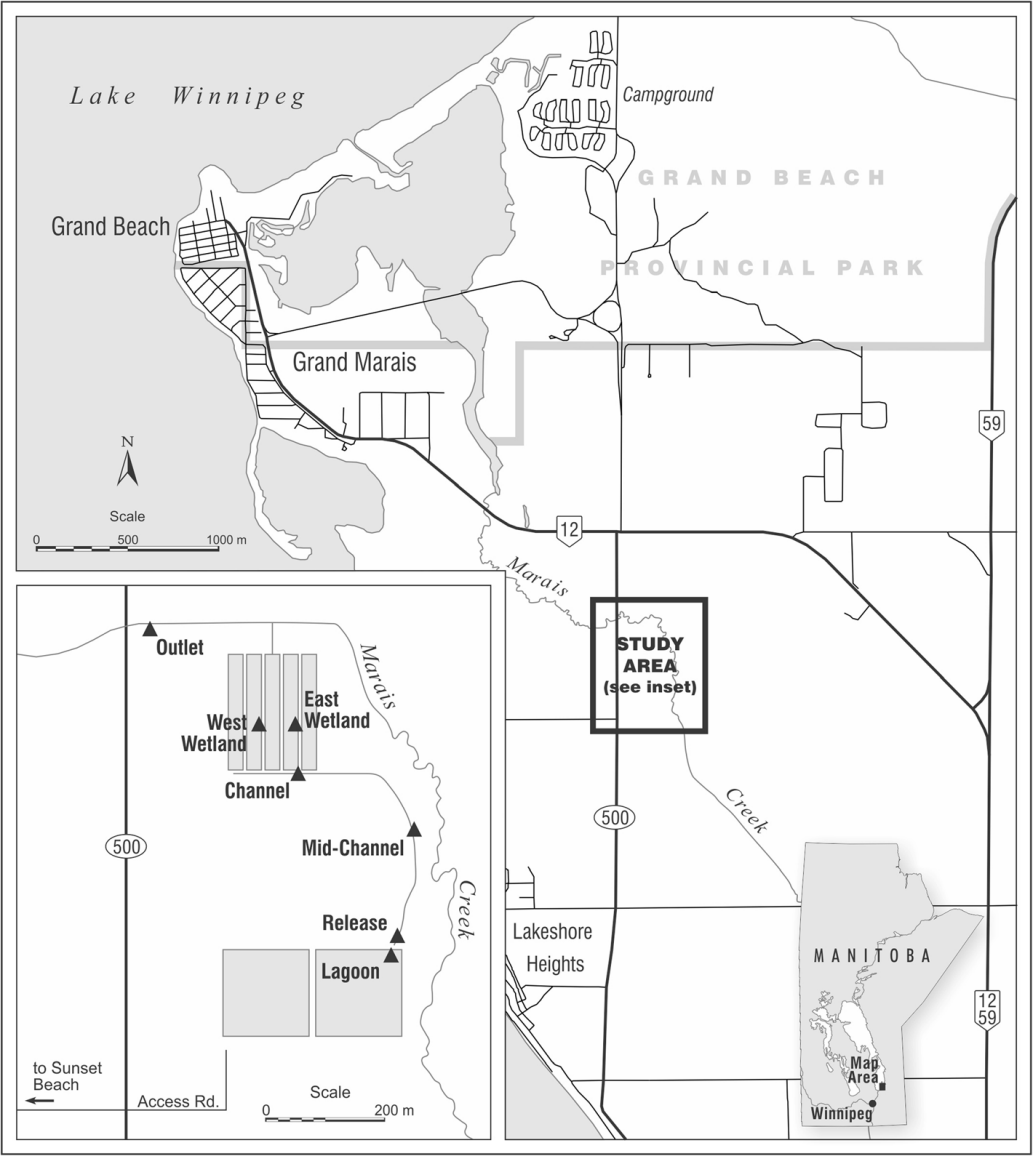
**Map showing the seven sampling site locations in the Grand Marais treatment system in Manitoba, Canada.** Sites were Lagoon, Release, Mid-Channel, Channel, East Wetland, West Wetland, and Outlet.

Samples were collected from the lagoon and from six sites within the treatment wetland between the influent entry point and the outlet into receiving surface waters. Upstream to downstream (direction of lagoon effluent flow), the sites were as follows: Lagoon, Release, Mid-Channel, Channel, East Wetland, West Wetland, and Outlet (Figure 1). Results of water quality monitoring at the seven sites in 2012 are reported in Table 1. The measured temperatures varied over the course of the sampling season, as expected, and among sites by as much as 5.3°C on the same sampling day. Conductivity was generally least at the Outlet site and greatest at the Lagoon or Release sites. Concentrations of chlorophyll-a (measured at ~ 30 cm below the surface) were quite variable among sites, with the greatest concentrations measured at the East Wetland, West Wetland, and Lagoon sites. In general, the concentrations of DO (dissolved oxygen) were quite low in the lagoon and wetland, with several measurements below 1 mg/L. The greatest concentration of DO was measured at the Release and Outlet sites, and the least concentration of DO was measured in the channel and lagoon. Measured pH ranged from 6.9 to 10.0 with the greatest pH values observed at the Lagoon, Release, and Channel. The Outlet and East Wetland sites typically had the lowest values of total suspended solids (TSS), and the Lagoon had the greatest values of TSS.

**Table 1:** Water quality parameters measured in sampling sites near the Grand Marais treatment wetland during 2012

**Date**	**Site**	**Total suspended Solids (mg/L)**	**Nitrite + nitrate (mg/L)**	**Total ammonia + ammonium (mg/L)**	**Total phosphorus (mg/L)**	**Chl A (μg/L)**	**DO (mg/L)**	**T (°C)**	**Conductivity (mS/cm)**	**pH**
**May 22**	Lagoon	29 ±3	0.14 ± 0.01	1.7 ± 0.1	3.1 ± 0.10	1.5 × 10^2^	1.5	15.3	1.1	NA
	Outlet	8.2 ± 0.8	<LOD	0.010	0.030 ± 0.002	12	7.4	14.8	0.42	NA
**June 15**	Lagoon	34 ± 6	<LOD	0.060 ± 0.01	1.5 ± 0.04	1.6 × 10^2^	1.5	18.1	1.1	9.25
	Outlet	8.6 ± 1	<LOD	0.020 ± 0.01	0.040 ± 0.01	15	7.6	17.5	0.38	7.56
**July 16**	Release	12 ± 0.8	<LOD	0.17 ± 0.01	0.68 ± 0.01	11	5.6	20.1	0.99	9.68
	Channel	12 ± 1	<LOD	0.22 ± 0.02	0.46 ± 0.01	22	0.60	19.4	0.89	9.26
	East Wetland	7.4 ± 2	<LOD	0.18 ± 0.04	0.40 ± 0.04	80	0.90	18.6	0.89	7.85
	West Wetland	12 ± 2	<LOD	0.030 ± 0.02	0.10 ± 0.04	1.7 × 10^2^	0.90	17.2	0.54	7.10
	Outlet	7.0 ± 1	<LOD	0.020 ± 0.001	0.010 ± 0.007	16	4.4	19.3	0.41	7.44
**July 23**	Release	9.7 ± 0.9	<LOD	0.25	0.39 ± 0.03	24	6.0	24.2	1.1	9.95
	Mid-Channel	31 ± 8	<LOD	0.14	0.61 ± 0.02	76	0.20	21.7	1.1	8.89
	Channel	13 ± 2	<LOD	0.040	0.51 ± 0.03	24	0.50	20.2	1.1	8.33
	East Wetland	5.3 ± 0.5	<LOD	0.060	0.10 ± 0.04	1.2 × 10^2^	0.30	19.5	1.1	7.31
	West Wetland	15 ± 5	<LOD	0.030	0.040 ± 0.01	1.3 × 10^2^	0.30	18.9	0.72	6.92
	Outlet	4.2 ± 1	<LOD	0.020	<LOQ	15	4.3	23.3	0.38	7.46

Measurements are presented as mean value ± SD; <LOD = below the limit of detection; <LOQ = below the limit of quantification; NA= not available; Chl A = chlorophyll-a; DO = dissolved oxygen. LODs: Nitrite + nitrate – 0.05 mg/L, Total ammonia + ammonium – 0.010 mg/L; LOQs: Phosphorus – 0.010 mg/L.

An approximate discharge rate was calculated using the distance from lagoon release to the Channel site. Assuming a discharge volume of 23,200 m^3^, discharge rate was ~0.02 m^3^/s, averaged over the course of the entire lagoon release period (July 11 to 24), and residence time within the length of the channel was approximately 20 hours. The channel itself is a ditch with wetland plants lining the sides. Residence time in the wetland was not determined due to the complexity of the flow patterns and the altered channels, which no longer followed the engineered ‘snaking’ flow pattern through winding rows. When the wetland was constructed in 1996, it was recommended that it receive inputs from the secondary lagoon in the fall (September 1 to October 31) with anticipated retention times of at least five to ten days.

### Concentrations of nutrients

The concentrations of nitrate + nitrite, total ammonia + ammonium, and total phosphorus are also reported in Table 1. Only one sample, from the Lagoon site, had a detectable and quantifiable concentration of nitrate + nitrite of 0.14 mg/L. Measurements of total ammonia + ammonium ranged from 0.02 to 1.7 mg/L. These measured concentrations were generally greatest at the Lagoon, Release, and Channel sites and least at the Outlet site. Finally, total phosphorus was measured between 0.01 and 3.1 mg/L, with the greatest concentrations occurring at the Lagoon site and the least concentrations at the Outlet site.

### Concentrations of pharmaceuticals and pesticides

Only six of the thirty-nine target pharmaceuticals and pesticides were detected in samples from the Grand Marais study area: the herbicides 2,4-D and atrazine, the anticonvulsant carbamazepine, the lipid regulator gemfibrozil, and the antibiotics sulfamethoxazole and sulfapyridine (Additional file 1: Table S1 for full list of compounds and LODs and Additional file 1: Table S2 for full list of concentrations observed). Attempts were made to determine dissipation rate constants for these compounds based upon collected field data. However, constants could not be calculated since consistent dissipation was not observed between sites along the channel, possibly due to insufficient retention time in the wetland. The range of concentrations measured for each compound and the differences among sites are discussed below. There were only two sampling events (June 15 and July 23/25) for which Polar Organic Chemical Integrative Sampler (POCIS) and solid phase extraction (SPE) samples could be compared quantitatively. The concentrations measured from POCIS samples were quite consistent with those measured by SPE, which is in agreement with previous comparisons of these techniques at similar sites in Manitoba [4]. This agreement suggests that the time-weighted-average concentrations, observed by POCIS, may likely be in line with the day-to-day fluctuations expected in a dynamic system, and thus are an integrator of changing temporal levels of chemicals with time [25]. It is important to note, however, that such agreement does not necessarily prove that time-weighted-average concentrations must be at the same concentration ranges as that of grab measurements, which could fortuitously measure chemicals at abnormally high or low concentrations.

**Figure 2:**
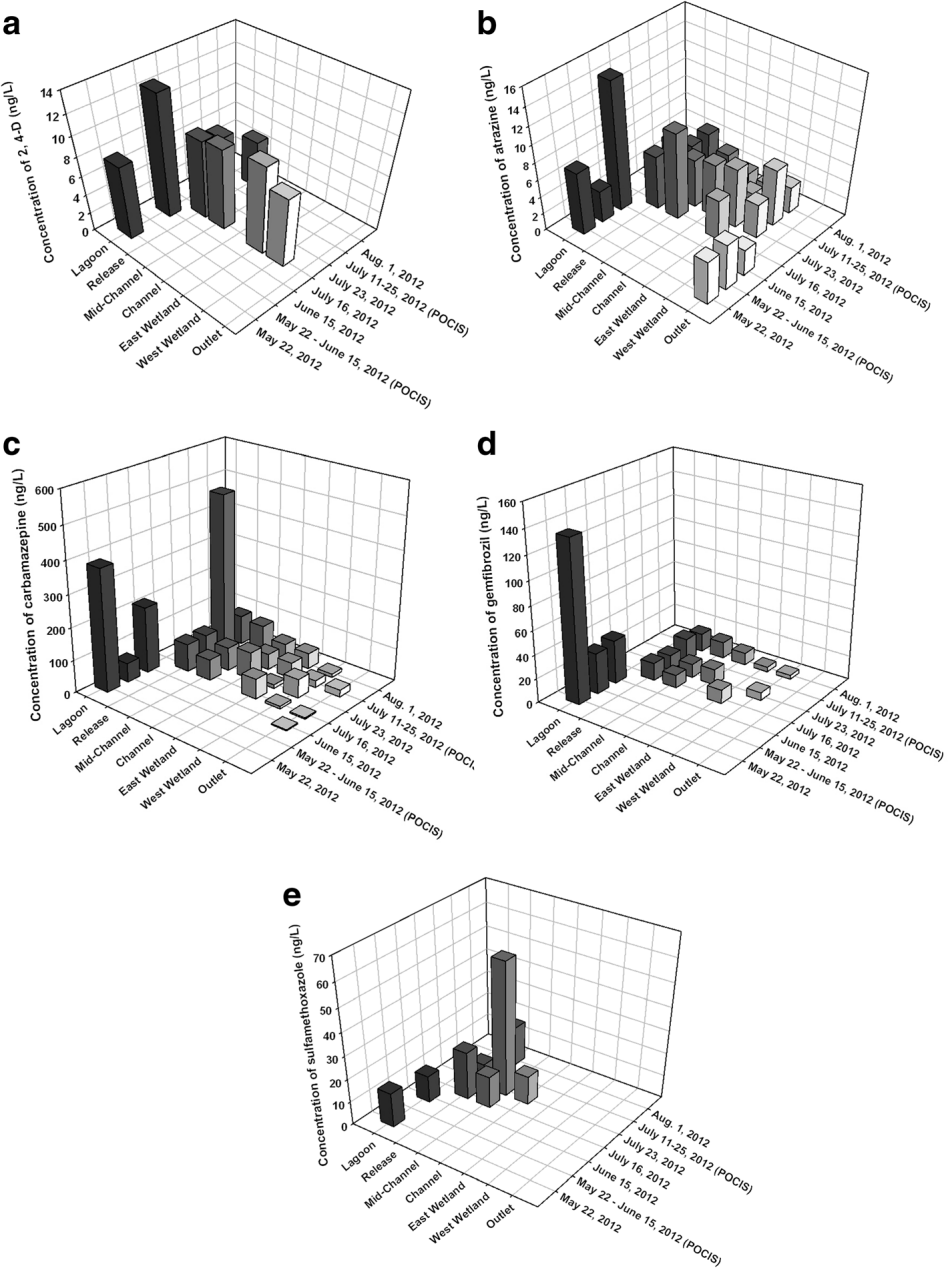
Mean concentrations of a) 2,4-D, b) atrazine, c) carbamazepine, and d) gemfibrozil and e) sulfamethoxazole measured at locations in the Grand Marais treatment wetland in summer 2012 by POCIS or SPE sampling.

In the majority of the water samples analyzed, 2,4-D was either not detected or below the limit of quantification (LOQ) (Figure 2a), similar to results observed elsewhere in rural Manitoba [4]. Most of the detections occurred on July 16, 2012, with very similar concentrations measured across the sites, in the range of 7 to 9 ng/L. The greatest concentration of 2,4-D measured was 13 ng/L at the Lagoon site using SPE. The Lagoon site had significantly more 2,4-D present than the Channel, West Wetland, or Outlet sites (p<0.05). There were no significant differences between concentrations of 2,4-D in the Channel and the Outlet (p>0.05), so elimination of 2,4-D was not significant within the wetland.

Concentrations of atrazine varied from non-detectable to 15 ng/L, with at least one detection in all sampling locations (Figure 2b). Atrazine was detected in the Lagoon and Outlet sites in the spring sampling and consistently in the wetland and channel during the summer months. There was a significant difference between the Channel site upstream and the Outlet site downstream of the wetland (p<0.05), suggesting that elimination processes occurred in the wetland.

The greatest concentrations of carbamazepine in individual samples were measured by POCIS at the Release site (500 ng/L) and by SPE at the Lagoon (380 ng/L) (Figure 2c). Generally, concentrations of carbamazepine were below 100 ng/L and detections were recorded for all sampling sites over the course of the study period. There was a significant reduction observed between entry and release points at the wetland (i.e. Channel and Outlet, respectively) (p<0.05), but there were no significant differences in concentrations of carbamazepine among any of the other sites. These results suggest processes within the wetland may significantly reduce concentrations of carbamazepine.

Gemfibrozil was detected at all sites except for the Outlet and the greatest concentration of 140 ng/L was measured by SPE at the Lagoon (Figure 2d). Concentrations of gemfibrozil were generally greater at the Release site than at the Wetland or Channel sites. The Lagoon site had significantly greater concentrations of gemfibrozil than any other sampling location (p<0.05), but there was no significant reduction in concentration observed as a result of passage through the treatment wetland (p>0.05).

Sulfamethoxazole was detected on five sampling days and only at four of the sampling sites: Lagoon, Release, Mid-Channel, and Channel (Figure 2e). The greatest concentration measured in an individual sample was 58.1 ng/L, which was measured at Mid-Channel by SPE. Statistical analyses found no differences among any of the sampling sites in terms of concentrations of sulfamethoxazole or between locations upstream and downstream of the wetland (p>0.05), indicating that elimination of sulfamethoxazole was not occurring within the Grand Marais treatment system.

Finally, sulfapyridine was only measured once at a quantifiable concentration (7.9 ng/L) and this was at the Outlet site. It was detected a few other times below LOQ, and the majority of samples had non-detection of sulfapyridine. There were no trends observed among sites for concentrations of sulfapyridine since it did not persist in the environment and was therefore not detected regularly in samples.

The hazard quotients (HQs) ranged from 3.2 × 10^-5^ to 1.5 × 10^-1^ (Table 2) so none of the pesticides or PPCPs quantified were deemed to pose a significant hazard (HQ> 1) to aquatic plants, invertebrates, or fish. The greatest HQ values were for gemfibrozil and sulfamethoxazole, calculated for fish and primary producers, respectively. Sulfapyridine, atrazine, and 2,4-D were expected to pose the least hazard to primary producers, invertebrates, and fish based upon the calculated HQs.

**Table 2:** **Calculated hazard quotients for pesticides and PPCPs detected**^**a **^**in the Grand Marais treatment wetland and surrounding sampling sites in 2012 (adapted from Carlson *et al., *2013)**[4]

**Compound**	**Species**	**Toxicity endpoint**	**Toxicity value (mg/L)**	**MEC**^**b **^**(mg/L)**	**HQ**^**c**^	**Reference for toxicity value**
**2,4-D**	*Ranunculus aquatilis* (Water buttercup)	EC50 – 4 week relative growth	0.2	1.3 × 10^-5^	6.4 × 10^-2^	Belgers *et al.,* 2007 [26]
	*Daphnia magna*	EC50 – 48 h immobilization	25	1.3 × 10^-5^	5.1 × 10^-4^	Martins *et al.,* 2007 [27]
	*Oncorhynchus mykiss* (Rainbow trout)	LC50 – 96 h exposure	100	1.3 × 10^-5^	1.3 × 10^-4^	Little *et al.,* 1990 [28]
**Atrazine**	*Lemna minor*	IC50 – 7 day growth inhibition	61.7	1.5 × 10^-5^	2.4 × 10^-4^	Teodorovic *et al*., 2012 [29]
	*Daphnia magna*	EC50 – 48 h immobilization	25.3	1.5 × 10^-5^	1.7 × 10^-2^	Phyu *et al.,* 2004 [30]
	*Oncorhynchus mykiss*	LC50 – 28 day exposure	0.87	1.5 × 10^-5^	5.8 × 10^-4^	Giddings*et al.*, 2005 [31]
**Carbamazepine**	*Lemna minor*	EC50 – 7 day growth inhibition	22.5	5.0 × 10^-4^	2.2 × 10^-2^	Cleuvers, 2003 [32]
	*Daphnia magna*	EC50 – 48 h immobilization	>100	5.0 × 10^-4^	5.0 × 10^-3^	Cleuvers, 2003 [32]
	*Oryzias latipes* (Japanese medaka)	LC50 – 48 h exposure	35.4	5.0 × 10^-4^	1.4 × 10^-2^	Kim *et al.,* 2007 [33]
**Gemfibrozil**	*Chlorella vulgaris*	LC50 – 24 h exposure	60	1.4 × 10^-4^	2.3 × 10^-3^	El-Bassat *et al.,* 2011 [34]
	*Daphnia* spp.	ECOSAR EC50 (acute)	6	1.4 × 10^-4^	2.3 × 10^-2^	Sanderson *et al.,* 2003 [35]
	Fish spp.	ECOSAR EC50 (acute)	0.9	1.4 × 10^-4^	1.5 × 10^-1^	Sanderson *et al.,* 2003 [35]
**Sulfamethoxazole**	*Pseudokirchneriella subcapita*	EC50 – 72 h growth inhibition	0.52	5.8 × 10^-5^	1.1 × 10^-1^	Isidori *et al*., 2005 [36]
	*Daphnia magna*	EC50 – 24 h immobilization	25.2	5.8 × 10^-5^	2.3 × 10^-3^	Isidori *et al*., 2005 [36]
	*Oryzias latipes*	LC50 – 96 h exposure	562.5	5.8 × 10^-5^	1.0 × 10^-4^	Kim *et al.,* 2007 [33]
**Sulfapyridine**	*Pseudokirchneriella subcapitata*	IC50 – 72 h growth inhibition	10.2	7.9 × 10^-6^	7.7 × 10^-4^	Blaise *et al*., 2006 [37]
	*Thamnocephalus platyurus* (Beavertail fairy shrimp)	LC50 – 24 h exposure	144.4	7.9 ×10^-6^	5.5 × 10^-5^	Blaise *et al*., 2006 [37]
	*Oncorhynchus mykiss*	48 h TEC – primary hepatocyte exposure	>249	7.9 ×10^-6^	3.2 × 10^-5^	Blaise *et al*., 2006 [37]

^a^ A full list of screened compounds and their limits of detection can be found in Additional file 1: Table S1.

^b^ MEC = Maximum environmental concentration measured in the current study.

^c^ HQ = Hazard quotient.

### Presence of ARGs

**Figure 3:**
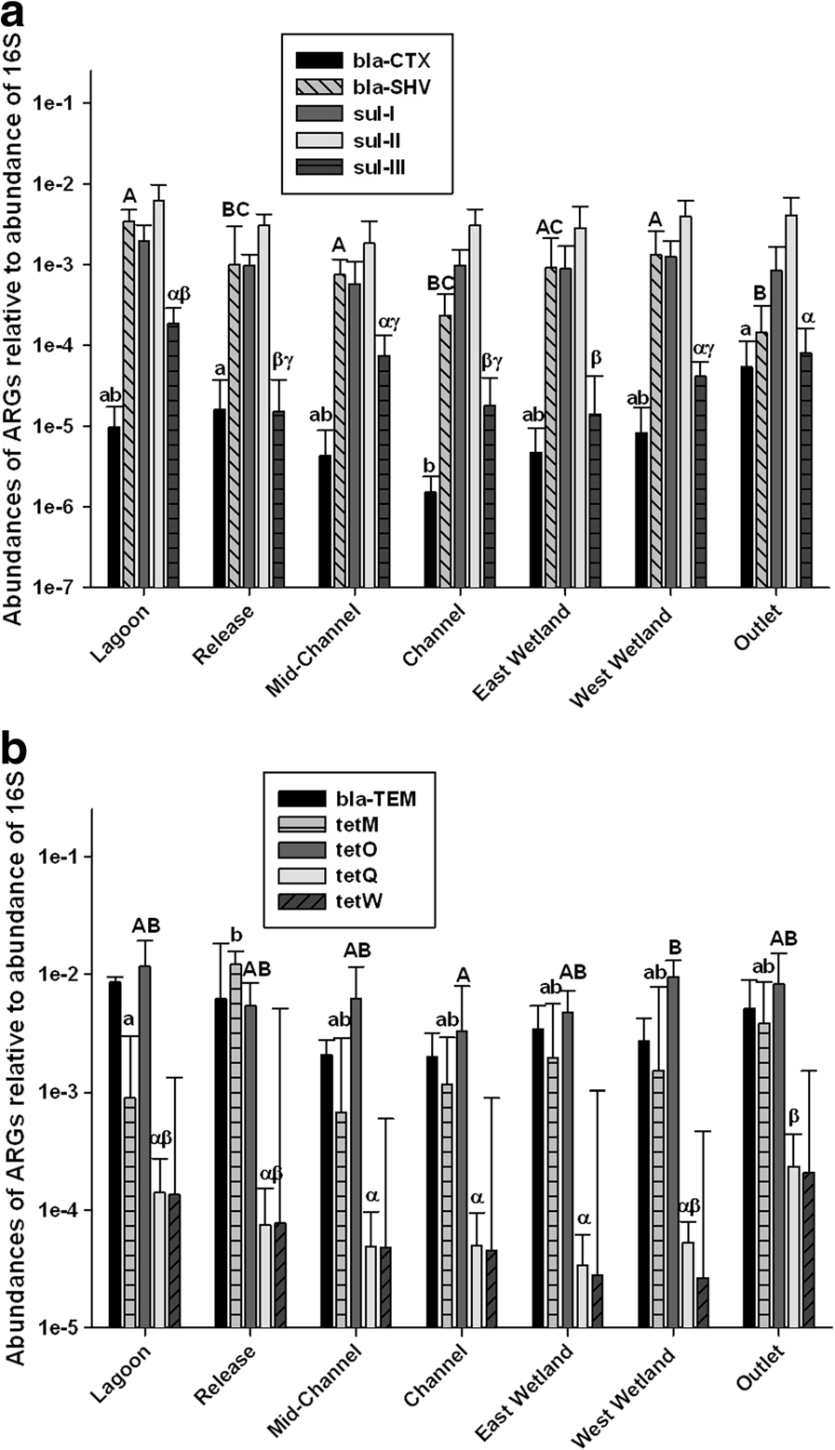
**a) Mean (±SD) abundances of *bla***_**CTX**_**, *bla***_**SHV**_**, *sul-*I, *sul-*II, and *sul-*III antibiotic resistance genes standardized to abundances of 16S-rRNA from samples collected at locations in the Grand Marais treatment wetland in summer 2012 and analyzed using qPCR.** Statistically significant differences (p<0.05) in abundances of individual genes are indicated using different lower case, upper case, and Greek letters. **b**) Mean (±SD) abundances of *bla*_TEM_ and *tet*^r^ antibiotic resistance genes standardized to abundances of 16S r-RNA from samples collected at locations in the Grand Marais treatment wetland in summer 2012 and analyzed using qPCR. Statistically significant differences (p<0.05) in abundances of individual genes are indicated using different lower case, upper case, and Greek letters.

Abundances of 16S rRNA genes (a surrogate measure of total bacteria) were fairly consistent over time at each site, with values ranging between 10^5^ and 10^7^ genes per mL of water sampled (Additional file 1: Table S3). Abundances of ARGs were standardized to the abundance of 16S in each sample to provide an indication of the proportion of the bacterial genes that could impart microbial resistance (Figure 3a and 3b). All of the ARGs of interest were measured at each site and during every sampling event, except for *tet*(W) at the Release and Channel sites on August 1 and *bla*_SHV_ at the Outlet site on June 19. The *tet* gene series confers resistance to tetracycline, which includes ribosomal protection proteins and efflux pumps. The *bla* genes are for enzymes that provide beta-lactam resistance, with *bla*_TEM_ being most commonly found. *Sul* are genes for sulfonamide resistance.

**Figure 4:**
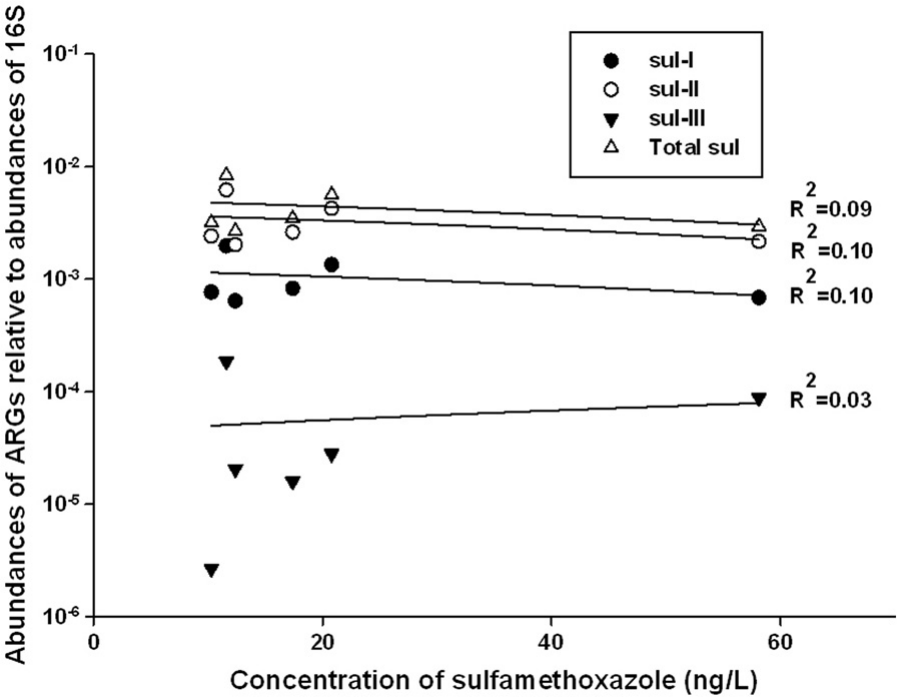
**Abundances of sulfonamide resistance genes (*sul-*I, *sul-*II, *sul-*III, and sum of all three) compared to concentrations of sulfamethoxazole measured in the Grand Marais treatment wetland in summer 2012.** There were no significant correlations between the abundances of ARGs and the concentration of antibiotics in the water (p>0.05).

Of the ten ARGs investigated in this study*,* the third multi-plex *tet*-gene series, (*tet*(K, L, M, O, S)) and *bla*_TEM_ generally had the greatest abundances in the samples from the Grand Marais treatment system. There was no obvious pattern of abundances of ARGs with movement upstream to downstream in the system, which did not warrant investigating individual determinants, but often the least measured abundance of ARGs was in the channel (Figure 3a and 3b). Concentrations of sulfonamide compounds were compared to abundances of *sul-*I*, sul-*II, and *sul-*III, but there was no significant linear relationship between abundances of these ARGs and measured concentrations of sulfonamides in the Grand Marais system (Figure 4). This is not surprising, as drug concentrations are below the Minimum Inhibitory Concentration (MIC) for most bacteria [38], and residence times are too short to monitor any effects at sub-inhibitory concentrations [39]; Most importantly, antibiotic resistance develops in the guts of treated organisms and therefore has different fates than the chemical antibiotic once released into the environment. Due to analytical issues, it was not possible to measure the concentrations of beta-lactam or tetracycline antibiotics in the system, so comparisons between those compounds and abundances of corresponding ARGs were not possible.

There was significant removal of *bla*_SHV_ between West Wetland and Outlet (p<0.05), but none of the other antibiotic resistant bacteria were significantly removed by the wetland. Overall, the abundance of each of the ARGs was less than 1% of the abundance of 16S genes, suggesting less than 1% of the bacterial population had the potential for resistance via one particular gene, which is typical for many lagoon systems, but the presence of multiple ARGs within a bacterium is also possible [40].

## Discussion

### Presence and removal of nutrients

Performance of the Grand Marais treatment wetland system was comparable to other wetlands, particularly in Europe, where some removal of nutrients (typically 30 to 50% of N and P) is expected, assuming loadings are not excessive [41]. Concentrations of phosphorus measured in the wetland were consistent with previous studies of other wetlands from the Interlake region of south-central Manitoba [42] and were below trigger levels for all lake types. Therefore, phosphorus was not considered a hazard for aquatic organisms. Nitrate and nitrite were not a concern at any of the sites sampled as they were only detected in one sample during the entire study duration. There were several instances where concentrations of total ammonia + ammonium surpassed the Canadian regulations to protect aquatic life, as specified by the particular pH and temperature conditions during the time of sampling [43]. Excess total ammonia + ammonium was measured in both the channel and in the lagoon and may be a result of processes within the treatment system whereby anoxic conditions in stagnate water can produce ammonia [44]. The elevated ammonia in the lagoon appeared to be more transient than that in the channel since several consecutive samples from the Channel and Mid-Channel sites had excess ammonia. However, concentrations of total ammonia, calculated according to Canadian wastewater regulations [45], did not surpass the requirements for wastewater.

As mentioned above, pH played a role in the allowable concentration of ammonia, and there were several instances where pH was measured above levels that are recommended for fresh water organisms (i.e. > 9.0) [43]. However, measured pH and conductivity in the Grand Marais wetland were very similar to other wetland areas in the Rural Municipality of St. Clements [46]. The DO levels were quite low in both the lagoon and the wetland system (except for the Outlet site) and generally were well below the recommended guidelines for freshwater habitats (i.e. <5.5 mg/L) [43]. The East Wetland and West Wetland sites had concentrations of DO that were below those typically measured in other Manitoba wetlands. However, DO at the Outlet site was consistent with concentrations measured in other local wetlands [42,47]. The Canadian wastewater regulations for TSS require concentrations no greater than 25 mg/L for a short-term duration, and all measured values were at or below that level so TSS was not a concern in this system [45].

In general, concentrations of nutrients decreased from upstream of the wetland to downstream, indicating that the treatment wetland system was attenuating concentrations of nutrients in wastewater. In addition, many of the measured water quality parameters improved with movement from the lagoon to the outlet, so the wetland represented a fairly effective means of secondary treatment for municipal wastewater produced by small communities. The East Wetland had consistently greater concentrations of nutrients and conductivity than the West Wetland. This result was consistent with the longer travel time to the West Wetland than the East Wetland and greater opportunity for removal of excess nutrients. Although the retention time within the wetland was shorter than originally intended, as discussed further in the site description, a large-scale reconfiguration would not be necessary to meet standards for nitrate, nitrite, or TSS. However, modifications to the current operation and configuration should be considered in order to improve the pH, DO, and ammonia in the system. These parameters should continue to be monitored since they were measured at levels of concern over the course of the study.

### Presence and removal of pesticides and PPCPs

The presence or absence of specific micropollutants is partly attributable to the residence time within septic tanks prior to entry into the sewage lagoons. While photodegradation cannot occur in septic tanks, other degradative processes such as anaerobic microbially-mediated biotransformation likely do occur. Consequently, relatively labile compounds such as naproxen and diclofenac [48] were probably degraded to some extent, and possibly below detection limits prior to arriving at the lagoons. Sorption of analytes to septic tank particulates is also likely. The two sulfonamides are photolabile, so photolysis within the sewage lagoon could also have contributed to the resulting non-detection in the majority of samples. On those occasions where sulfamethoxazole or sulfapyridine were detected, it may have been a result of light attenuation and shielding due to turbidity and dissolved organic carbon (DOC) within the lagoon cells [49]. Atrazine and 2,4-D were measured at very low concentrations (typically <10 ng/L). Since only 2,4-D has been reported as applied agriculturally (at very low total loadings) in the municipality [50], the presence of atrazine was possibly due to use on private residential properties.

All detected and quantifiable micropollutants were measured in the ng/L range in samples from the Grand Marais lagoon and treatment wetland. There was a distinct spike in concentrations of micropollutants downstream of the lagoon during discharge and a subsequent reduction in concentrations with time. However, concentrations for some of the compounds, including carbamazepine and gemfibrozil, remained well above pre-discharge levels as of August 1 (nearly a week post-release), indicating that with the cease in flow from the lagoon, there is likely residual wastewater stagnating within the wetland. It is possible that some changes in concentration may be the result of water evaporation or addition; however, concentration and dilution effects would affect all analytes equally, which was not observed. While no measurements of pesticides and PPCPs occurred in winter, we note that these shallow wetland and stream systems are predominantly or completely frozen over the winter. This would presumably result in no removal of analytes by either microbial activity or photodegradation (i.e., light penetration would be prevented almost completely by ice cover and would be of low intensity in any event) until spring melt.

A hazard assessment was conducted using the maximum concentration of each compound measured in the environment and comparing it to toxicity thresholds for aquatic plants, invertebrates, and fish (Table 2). While none of the calculated HQs surpassed a value of unity, those for gemfibrozil and sulfamethoxazole approached the threshold of concern for fish and aquatic plants, respectively, so these compounds might warrant more regular monitoring.

There was significant removal of atrazine and carbamazepine by the treatment wetland, but the wetland did not significantly or consistently attenuate concentrations of 2,4-D, gemfibrozil, or sulfamethoxazole. Due to the very infrequent measurement of sulfapyridine above the limits of detection or quantification, it was not possible to determine the impact of the wetland on this compound. In general, concentrations of these compounds decreased from upstream to downstream, lagoon to outlet, but there was no evidence for significant elimination within the wetland itself.

In previous studies, removal of atrazine within wetlands was dependent upon retention time [51]. Kadlec and Hey [51] reported between 25 and 95% removal of atrazine in different wetland cells after 3 to 4 weeks of retention time. Similarly, Kao et al. [52] observed up to 99% removal of atrazine within 15 days in anaerobic cells spiked with sucrose media, but less than 9% removal in control wetlands that were not inoculated with media or a nitrogen source. While removal of atrazine from wastewater can be quite variable and very dependent upon the specific substrates and characteristics of the wetland, the results from the current study suggest that the Grand Marais wetland conditions are conducive to removal of atrazine.

Previous studies have reported relatively effective removal of carbamazepine, with 51% removal of carbamazepine via treatment in a forested wetland for 27 days, and up to 80% removal of carbamazepine in *Typha-*inhabited freshwater wetlands over the course of 6 days [1,21]. These results agree with those of the current study where lagoon discharge was treated in a *Typha*-dominated wetland with a residence time of approximately 20 hours. While carbamazepine is relatively persistent, it may be removed to some extent by sorption to suspended particles and uptake by plants, including biotransformation by *Typha* spp. [53,54]. That having been said, sorption is unlikely to be a significant removal process for the analytes that were consistently detected. For example, over 99% of carbamazepine is expected to be in the dissolved phase, given the octanol-water partition coefficient of 10^2.45^[55] of the neutral species (predominant at our observed pH values, Table 1) and the maximum observed suspended matter in the lagoon/wetland system (TSS of 29 mg/L, Table 1), assuming all of this matter is organic carbon. While polar organic chemicals can also sorb by other mechanisms, e.g., ion exchange, the low particulate levels observed preclude the likelihood that sorption to such is a major attenuation process, at least in this system.

Unlike the current study, Conkle et al. [1] noted >90% removal of sulfonamides and 95% removal of gemfibrozil, however, the differences may have been a result of the 27 day retention period. In a comparative study, treatment wetlands were found to be ineffective for removal of sulfamethoxazole [18], so removal of this class of PPCPs may be site-specific. Microbial degradation of gemfibrozil has been observed to be relatively rapid in groundwater conditions [56], and in the current study, there was a general pattern of reduction in concentration with passage through the wetland. The lack of statistical significance might be due to the small number of samples collected at the Channel site or the relatively low concentrations found following release from the lagoon.

### Presence and removal of ARGs

Similar abundances of sulfonamide resistance genes were measured in a previous study of a river impacted by both urban and agricultural impacts [57]. Sulfonamides are used in both human and veterinary medicine and target the enzyme dihydropteroate synthase (DHPS), which is part of the folic acid pathway [57]. A previous study reported normalized abundances of sulfonamide resistance genes between 0.02 and 7.7% [12], which agrees with the findings in the Grand Marais system (~0.5%). The sulfonamide resistance genes assessed in the current study (*sul*-I, *sul*-II, and *sul*-III) were measured at relatively high concentrations compared to other ARGs. However, since there was no significant relationship between concentrations of sulfonamides and abundances of sulfonamide resistance genes (Figure 4), presence of these genes within the wetland are probably well established as a result of repeated inputs into the system, both past and present. Concentrations of other types of antibiotics (e.g. tetracyclines, beta-lactams) were not measured, so it is unclear whether there was any cross-resistance within the system as a result of the presence of those specific compounds.

Tetracyline resistance genes (e.g. *tet*(M), *tet*(O), *tet*(Q), and *tet*(W)) have been investigated in other studies due to their common transmission in the environment [58] and these ARGs had relatively great abundances in the current study. Smith et al. [58] measured abundances of ARGs in cattle feedlot lagoons and reported concentrations of tetracycline resistance genes ranging from approximately 10^4^ to 10^6^ copies per mL, which is within 1 or 2 orders of magnitude of the concentrations measured in the current study. The abundances of tetracycline resistance genes measured by Pei et al. [57] were 2 to 3 orders of magnitude less than those measured in the current study. Some of the differences in abundances may be due to sampling in sediments by Pei et al. [57] rather than in water, as in the current study.

There were no obvious trends when upstream (i.e. lagoon) and downstream (i.e. output) abundances of ARGs were compared. The only ARG for which the relative abundance was significantly less at the output than in the treatment wetland was *bla*_SHV_. There may have been some removal of microbes bearing this gene in the wetland, but none of the other ARGs were significantly reduced by treatment with the wetland. Previous studies with full-scale and bench-scale wetlands have demonstrated significant removal of bacteria from wastewater, resulting in an approximate reduction of two orders of magnitude or up to 99% of bacteria [40,59]. However, Vacca et al. [59] noted that removal efficiency was highly dependent upon the operation conditions of the wetland, as well as the presence of plants. Removal of bacteria from the Grand Marais treatment wetland likely occurred via a combination of filtering by those plants that were present and sedimentation since DO levels were insufficient in many sites to promote predation by micro-invertebrates [41,59].

With the qPCR method of quantifying abundances of genes within samples from a system, genes from both living and dead bacteria are included so the results may not necessarily represent the true proportion of living bacteria that might be resistant to antibiotics [57]. This should be taken into consideration when quantificatiying of abundances of ARGs within the system. While the Grand Marais treatment wetland appeared to remove bacteria in general, there was no indication that there is any targeted removal of ARGs in the wetland. As a result, the Grand Marais treatment wetland does not appear to be an optimal system for removal of ARGs in its current operational state.

## Materials and methods

### Study area

The Grand Marais treatment wetland (50° 31’ N and 96° 35’ W) is located in the Rural Municipality of St. Clements, near Grand Marais, MB, and Lake Winnipeg (Figure 1). The wetland receives rural wastewater from the secondary lagoon of a two-lagoon system located directly to the south. Each lagoon is approximately 134 m by 134 m and 2.3 m in depth, with a total storage volume of 29,400 m^3^ and licensing to allow up to 1.5 m of liquid within the lagoon cells [60]. There are no direct sewage lines into the lagoon facility, so sewage is aged for an unknown length of time in septic tanks before hauling by septic trucks to the lagoon. Consequently, retention time within the primary lagoon cell is also not well defined. While time within the secondary cell is better known, understanding the residence times in the lagoons was not central to this study since the wetland performance was the main area of focus, though determining this would help to better understand inter-year variability. Prior to the 2012 release, the last release event was July 2011, meaning some waste had aged a maximum of approximately one year in the secondary lagoon.

The treatment wetland is composed of a 0.7 km long wetland channel from the lagoon to the five channel “rows”; the rows collect discharged lagoon water from the channel and direct it through the wetland. The five rows were intended to achieve a ‘snaking’ configuration whereby water would enter the wetland at a single point and exit after passing through all of the rows. The wetland was designed to retain water at a depth of 15 to 30 cm throughout the year. Prior to release, the wetland contained water, which would have been inputted from snow melt, precipitation, and remaining effluent from the previous year. In reality, the residence time in the wetland is likely much shorter than originally anticipated (five to ten days). This is due to water entering the wetland via all of the rows and flowing directly through to Marais Creek as a result of the loss of the discrete rows since construction in 1996, and a lack of sufficient hydraulic head to maintain flow at the designed hydraulic residence time. Treated wastewater from the wetland ultimately flows into Lake Winnipeg. Lagoon water is released into the treatment wetland one or two times per year (i.e., summer, normally June or July, and fall, normally October) depending on lagoon capacity. This summer and possible fall release is typical of most lagoon systems in Manitoba [4]. The volume, frequency, and timing of releases have varied over recent years because the size of the primary lagoon cell has increased.

### Study sites

Sampling was performed both before and after lagoon release in 2012. There were a total of six sampling sites in the wetland, as well as one site in the secondary treatment lagoon (Figure 1). The six sites were selected at different locations within the treatment wetland between the influent entry point and the outlet into the surrounding water. The site names from upstream to downstream were as follows: Lagoon, Release, Mid-Channel, Channel, East Wetland, West Wetland, and Outlet.

The Release and Mid-Channel sites were dominated by submergent plants, as well as *Lemna* spp*.,* and had water depths of ~1 m. Emergent species, particularly *Typha* spp., and some small bushes dominated the East Wetland and West Wetland sites. West Wetland had a water depth of about 40 cm while East Wetland was about 60 cm deep. In the deeper areas of both wetland sites, *Lemna* spp. and several submergent species were present where the wetland water levels are sustained during dry years [60]. The Outlet site was relatively deep (~1-1.5 m deep, depending upon precipitation and evaporation) and wide (2 m wide at culvert) compared to the other sites thus resulting in greater flow. No submergent or emergent wetland plant species were present at the Outlet, but there were grasses and other terrestrial vegetation growing along the creek bank. The hydrology of Marais Creek (which receives flow from the Outlet) is not defined due to a lack of gauging stations, but it is ~3 m wide and discharge of the creek has been measured at 0.06 m^3^/s [60].

### General water quality parameters

General water quality and physico-chemical parameters (dissolved oxygen (DO), conductivity, chlorophyll-a, pH, and water temperature) were measured during each sampling event using a YSI 6600 Multi Parameter Water Quality Meter sonde (YSI Inc., Yellow Springs, OH).

### Sample collection

Grab samples for nutrient analyses, total suspended solids (TSS), ARGs, and PPCPs were collected on May 22, June 15, July 16, July 23, and August 1. All sample types were collected on each sample day with the exception of: August 1, where samples were only taken for PPCP analysis and ARGs, and May 22, where no antibiotic resistance genes samples were taken. Summer release from the lagoons into the treatment wetland occurred from July 11 to 24, 2012. Prior to release (May 22 and June 15), samples were taken in the lagoon and at the Outlet site, and during and after release (July 16 and 23, and August 1) samples were taken in the treatment wetland.

Samples were collected using sterile 500 mL polyethylene bottles and 4 L amber glass bottles, as required for the analytical procedures. Each bottle and cap was rinsed three times with sample water and the rinsate was discarded downstream from the sampling location. The bottle was then lowered into the water to a depth of approximately 30 cm below the water surface, filled, and capped underwater with care taken to ensure no headspace was left in the bottle. Extra sample bottles filled with nanopure (18 MΩ cm) Milli-Q water (Millipore Corporation, Billerica, MA) were opened at the sampling sites to serve as field blanks. During the wastewater release event, all equipment and the exteriors of sample bottles were disinfected after contact with wetland water using either isopropanol or bleach. Following collection, samples were stored at 4°C for up to 24 h for ARG samples and for 24-48 h prior to analysis of nutrients or extraction by solid phase extraction (SPE) prior to further analytical analysis for PPCPs. Extracted samples were stored at -20°C for no more than 6 weeks prior to analysis by LC/MS [61].

In addition to grab samples, Polar Organic Chemical Integrative Samplers (POCIS) (Environmental Sampling Technologies, St. Joseph, MO) were used for continuous time-weighted-average passive sampling of pharmaceuticals, as described in detail previously (refer to [4]). POCIS samplers were deployed at the lagoon and wetland outlet sites prior to release in 2012 (from May 22 to June 15), and at five wetland sites during release in 2012 (from July 11 to July 25). Samplers were prepared prior to deployment as described by Carlson et al. [4] and transported to each site in pre-cleaned containers filled with Milli-Q water. They were then suspended near the bottom of the river, wetland, or lagoon using aircraft cable tethered to rebar stakes. A triplicate set of POCIS samplers was deployed in each cage per sampling location for a 2-4 week period. After collection, samplers were rinsed with Milli-Q water, wrapped in foil that had been pre-ashed at 450°C, transported on ice, and frozen at -20°C for up to 2 months prior to extraction.

For extracted SPE samples and collected POCIS samples, minimal losses have been previously observed for the compounds of interest during frozen storage for 2-3 months (<7%) and up to 20 months (<20%) [61]. Therefore, any losses incurred during the storage period were deemed to be negligible and thus, no corrections were required to account for sample losses between collection and analysis.

### Nutrient and TSS analyses

Concentrations of nitrate + nitrite, total ammonia + ammonium, and total phosphorus were measured in the water samples. All nutrient analyses were performed by ALS Laboratory Group Analytical Chemistry and Testing Services (Winnipeg, MB), or in-house. Concentrations of nitrogen species were determined at ALS by flow injection analysis (Lachat Instruments, Loveland, CO), as per the manufacturer’s standard methods. The limits of detection (LOD) for ammonia and nitrate + nitrite were 0.050 mg/L and 0.010 mg/L, respectively. Total reactive phosphorus was measured in-house with a limit of quantification (LOQ) of 0.010 mg-PO_4_^3-^-P/L. Concentrations of phosphorus species were measured according to standard methods [62]. Total suspended solids (TSS) were quantified according to a modified procedure based on *Standard Methods for the Examination of Water and Wastewater*[62]*.*

### Pesticides and PPCP analyses

#### Analytical standards

A number of pharmaceutical classes were monitored, including estrogenic compounds, beta-blockers, antibacterial agents, antidepressants, NSAIDs, antibiotics, and lipid regulators. The specific compounds were selected due to their prevalence and/or persistence in the environment, based on published literature [63]. Analyses were conducting using analytical standards for thirty-nine pharmaceutical compounds and pesticides, with compounds and sources described in detail by Carlson et al. [4]. Tylosin and erythromycin standards were 97% and 95% pure, respectively, and all other chemicals were >98% purity. Stable isotope standards were >99% isotopically pure. Isotope sources are found in Carlson et al. [4]. A full list of the compounds and their LOQs can be found in Additional file 1: Table S1 of the online Supplemental Information.

#### Sample extraction

Grab samples from the lagoon and wetland were processed by solid phase extraction (SPE). Samples were sub-sampled into triplicate 500 mL samples (May 22, 2012) or 250 mL samples (all other dates), prior to filtration through 0.45 μm Metricel membrane filters (Pall Life Sciences, Mississauga, ON). A 25 ng aliquot of internal standard was added to each sample prior to extraction by 3 cc/60 mg OASIS™ HLB cartridges (Waters Corporation, Milford, MA). Samples were pre-conditioned with 2 mL of methanol, then 2 mL of water, and drawn through the cartridges at <5 mL/min. Cartridges were eluted with 3 mL of methanol at 0.5 mL/min. Extracts were evaporated under a stream of nitrogen at 40°C, reconstituted in 0.5 mL of 10:90 methanol:water, and filtered using a 0.22 μm polytetrafluoroethylene syringe filter (Restek Corporation, Bellefonte, PA). The final extracted volume was stored in darkness at 4°C for no longer than one week prior to analysis. One laboratory blank containing only Milli-Q water and internal standards and one field blank were extracted for each set of samples extracted by SPE.

POCIS samples were extracted by a similar method. Samplers were placed in Milli-Q water for 15 min to wet the HLB phase then were extracted in a 60 mL glass clean-up column containing 3-5 g of anhydrous sodium sulfate (Sigma, pre-dried at 450°C). Using 25-35 mL of methanol, individual POCIS sorbent was washed into the column and 50 ng of each internal standard was added to the solution. The extract was gravity-drained into a round bottom flask, and rotary-evaporated at 47-52°C to ca. 5 mL, then dried under a slow stream of nitrogen at 40°C. Samples were reconstituted in 0.5 mL of 10:90 methanol:water and filtered through a 0.22 μm syringe filter, then stored at 4°C for a maximum of one week before analysis. One laboratory blank POCIS, containing only the internal standards, and one field blank were extracted for each set of POCIS samplers.

#### Instrumental analysis

Concentrations of organic micropollutants were measured by liquid chromatography coupled with tandem mass spectrometry (LC/MS/MS). The standards and HPLC mobile phases were prepared using Milli-Q water and HPLC grade methanol (Fisher Scientific, Ottawa, ON) and buffered with 10 mM ammonium acetate (Sigma Aldrich, St. Louis, MO) or 90% formic acid (Fisher Scientific). Stock solutions of all micropollutants were prepared in HPLC grade methanol (Fisher Scientific). Details of the LC/MS/MS systems and their specifications have been described in detail previously [4].

External calibrations were performed using standards over a concentration range of 2-500 μg/L. Analytes were quantified using isotope dilution when possible, or via internal standardization [4]. Extraction efficiencies from SPE and POCIS extracts were 40-100%, but after correction with internal standards, based on spike-and-recovery experiments, efficiencies were 90-110% (data not shown). Relative standard deviations (RSDs) were <20% for triplicates from POCIS extractions and <8% for triplicates from SPE extractions. Concentrations of individual compounds were calculated using literature values for standard POCIS sampling rates [4]. In cases where these were unavailable, such as for diazinon, an average sampling rate for a suite of twenty-nine other pesticides and pharmaceuticals was used [63].

### Antibiotic resistance genes

#### Sample preparation

Prior to sampling, 500 mL polypropylene bottles (Chromatographic Specialties Inc., Brockville, ON) were autoclaved at 121°C for 2 h and capped until time of sampling. Samples for ARGs were collected as described above and stored for no more than 24 h at 4°C before extraction. Each ARG sample was filtered using a sterile, disposable Nalgene cup with a pre-installed 0.2 μm filter (Thermo Fisher Scientific Inc., Waltham, MA). The filter was removed using flame-sterilized forceps, folded, and placed into a 1.5 mL polypropylene centrifuge tube. The centrifuge tube was stored frozen at -20°C, and shipped on ice to the University of Strathclyde (Glasgow, UK) for analysis.

#### DNA extraction

A PowerSoil DNA Isolation Kit (MoBio Laboratories Inc., Carlsbad, CA) was used for DNA extraction. Filters were digested in a buffered solution with sodium dodecyl sulfate (SDS), which was provided by the kit. Cell disruption was achieved by a FastPrep24 instrument run twice for 20 s at a setting of 6.0. The remaining chemical precipitations and centrifugation procedures followed the manufacturer’s protocols. The DNA was eluted with molecular-grade DNase- and RNase-free water and stored at -80°C until further analysis.

#### Quantitative PCR

Abundances of 16S rRNA and ten ARGs were quantified by quantitative PCR (qPCR) using the Bio-Rad SsoFast™ EvaGreen® reagent system (Bio-Rad Laboratories Ltd., Mississauga, ON). The genes of interest were: *sul*-I*, sul-*II*, sul-*III (sulfonamide resistance genes), a series of multiplex primers for tetracycline resistance ([64], Additional file 1: Table S3), *bla*_CTX_, *bla*_TEM_, *bla*_SHV_ (beta-lactam resistance genes), and 16S-rRNA (a surrogate measure of total bacteria). A reaction with total volume of 10 μL was set up by adding 1 μL of DNA to 5 μL of SsoFast reagent and appropriate primers (from [65]) at 500 nM concentrations, and topping up with molecular-grade water. The Bio-Rad iQ5 was run for 2 min at 95°C for DNA denaturation, followed by 40 cycles at 95°C for 5 s, annealing temperature for 10 s (Additional file 1: Table S3), and 72°C for 10 s for DNA elongation. Reactions were monitored continuously by tracking the intensity of fluorescence.

Serially diluted plasmid DNA of known quantity was used for reaction standards and run in all reactions. Molecular-grade water was used as a reaction negative control. All standards and blanks were run according to the same procedures as the samples. For quality control purposes, a portion of the samples were selected at random and spiked with standards to assess reaction efficiencies. In addition, post-analytical melt curves from 55°C to 95°C were used to verify reaction quality. Abundances of genes are presented as log-transformed values, and were normalized to 16S-rRNA values to represent resistance per total bacteria.

### Hazard assessment

Hazard quotients (HQs) were calculated for each micropollutant of interest using standard tests and endpoints for aquatic toxicity assays, specifically those for primary producers, invertebrates, and fish. Briefly, estimates of effective concentrations (EC50) or lethal concentrations (LC50) were obtained from the appropriate literature. A predicted ‘no effect concentration’ (PNEC) was estimated for each target compound by dividing the lowest EC50 or LC50 by an uncertainty factor of 1000 [66]. The greatest measured environmental concentration (MEC) was then divided by the PNEC to obtain the HQ. Quotients less than 1 were considered unlikely to pose a concern, while those greater than 1 were considered to be of possible concern [67].

### Statistical methods

The experimental unit used was the individual sample or subsample and data is presented as mean ± standard deviation (SD) unless otherwise indicated. All analyses were conducted using SigmaStat (version 3.5, Systat Software, Inc.). Statistical differences between concentrations of pharmaceuticals at each sampling location, as measured by SPE and POCIS, were determined by two-way ANOVA tests followed by Holm-Sidak post-hoc tests where either raw or transformed data met the assumptions of normality and equality of variance. Concentrations of pharmaceuticals upstream (Channel) and downstream (Outlet) of the treatment wetland were compared using Student’s t-tests or Mann-Whitney tests.

Abundances of ARGs were standardized relative to abundance of 16S, whereby relative abundance of a particular ARG was equal to ‘log (ARG/16S)’. The relative abundances were then compared by two-way ANOVA tests followed by Holm-Sidak post-hoc tests where log-transformed data met the assumptions of normality and equality of variance. Where data did not meet the assumption of normality, Kruskal-Wallis ANOVA by Ranks tests were used and followed by Dunn’s post-hoc tests. Differences were considered significant at p<0.05.

## Conclusions

In the current study, there was a clear nutrient and micropollutant pulse into the treatment wetland as a result of lagoon release. The Grand Marais treatment wetland removed nutrients, suspended solids, and several pharmaceutical compounds. However, in its current configuration, it was not an effective treatment for most of the micropollutants that were quantifiable within the system or for removal of ARGs. Micropollutants were degraded with time and movement through the system and there was some reduction in bacterial counts from upstream to downstream. However, our results suggest that treatment wetlands operating in a manner similar to that of Grand Marais, and found in conditions akin to the Canadian Prairies, may not be optimal approaches for treating wastewater with detectable concentrations of micropollutants. The retention time within the current configuration of the Grand Marais wetland is shorter than originally designed. Therefore, upgrading the system to extend the retention time (e.g. fixing and cleaning out the channels to promote ‘snaking’) may be required to specifically target micropollutants and ARGs using these types of treatment systems.

## Abbreviations

ANOVA: Analysis of variance; ARGs: Antibiotic resistance genes; DO: Dissolved oxygen; EC50: Half maximal effective concentration; HQ: Hazard quotient; LC50: Half maximal lethal concentration; LOD: Limit of detection; LOQ: Limit of quantification; MEC: Maximum environmental concentration; PNEC: Predicted no effect concentration; PPCPs: Pharmaceuticals and personal care products; SD: Standard deviation; SPE: Solid phase extraction; POCIS: Polar organic chemical integrative sampler.

## Competing interests

The authors have no competing interests to declare.

## Authors’ contributions

JCA completed data analysis and drafted the manuscript. JCC collected samples using SPE and POCIS and analyzed samples by LC/MS. JKC and JEL collected samples and monitoring data and assisted in preparation of the manuscript. CWK analyzed the ARG samples. CSW and MLH devised the study, secured funding, and supervised personnel during sampling, analysis, and manuscript preparation. All authors read and approved the final manuscript.

## Supplementary Material

Additional file 1 The following additional data are available with the online version of this paper as supplementary information: additional details of the concentrations of micropollutants measured at each site, and detailed information on PCR conditions. Click here for file
