# Supplementary material for: Performance of a constructed wetland in Grand Marais, Manitoba, Canada: Removal of nutrients, pharmaceuticals, and antibiotic resistance genes from municipal wastewater
Source: Chem Cent J. 2013 Mar 18;7:54. doi: 10.1186/1752-153X-7-54 (PMC3610202; doi:10.1186/1752-153X-7-54)
Supplement: Additional file 1 — The following additional data are available with the online version of this paper as supplementary information: additional details of the concentrations of micropollutants measured at each site, and detailed information on PCR conditions. [file 1752-153X-7-54-S1.docx]

Performance of a constructed wetland at Grand Marais, Manitoba, Canada, in removing nutrients, pharmaceuticals, and antibiotic resistance genes from municipal wastewater

Julie C. Anderson^a^, Jules C. Carlson^a,b^, Jennifer E. Low^a^, Jonathan K. Challis^a,c^, Charles S. Wong^a,c^, Charles W. Knapp^d^ and Mark L. Hanson^b^*

^a^Richardson College for the Environment, Department of Environmental Studies and Sciences and Department of Chemistry, The University of Winnipeg, Winnipeg, MB, R3B 2E9, Canada

^b^Department of Environment and Geography, University of Manitoba, Winnipeg, MB, R3T 2N2, Canada

^c^Department of Chemistry, University of Manitoba, Winnipeg, MB, R3T 2N2, Canada

^d^David Livingstone Centre for Sustainability, Department of Civil & Environmental Engineering, University of Strathclyde, Glasgow, Scotland, G1 1XN

Supplementary Information

Table S1: Precursor and product masses with analysis method, limits of quantification, and source conditions for both the Applied Biosystems Q-Trap 2000 LC/MS/MS system and the Agilent 6410B LC/MS/MS system (Adapted from Carlson *et al*., 2013). Isotope dilution was used for compounds with an internal standard with a C_13_ or D label. All internal standards are labeled with an asterisk. In cases where a C_13_ or D labeled equivalent was not available, matched analytes and internal standards are given by matching superscripted numbers. Analytes whose concentrations were determined by external calibration are marked with a †.

|  |  |  |  |  | **Q-Trap** | | | | **Agilent MS** | | | |
| --- | --- | --- | --- | --- | --- | --- | --- | --- | --- | --- | --- | --- |
|  |  |  |  |  | **Matrix LOQs**  **(ng/L-H_2_O)** | | **Decl. Potent. (V)** | **Collision Energy (V)** | **Matrix LOQs**  **(ng/L-H_2_O)** | | **Fragmentor Voltage**  **(V)** | **Collision Energy**  **(V)** |
| **Compound** | **LC**  **Method** | **Precursor** | **Quantifier Product** | **Qualifier Product** | **POCIS** | **SPE** |  |  | **POCIS** | **SPE** |  |  |
| Atenolol | 1 | 267.2 | 145.2 | 190.0 | 18.0 | 18.0 | +52 | 35 | 2.4 | 2.0 | +135 | 16/16 |
| Atenolol-d_7_* | 1 | 274.2 | 145.1 |  | 18.0 | 18.0 | +52 | 35 | 2.4 | 2.0 | +135 | 16 |
| Atrazine | 1 | 216.1 | 174.1 | 146.2 | 9.0 | 6.0 | +60 | 25 | 2.4 | 3.0 | +130 | 16/20 |
| Atrazine-d_5_* | 1 | 221.1 | 179.1 |  | 9.0 | 6.0 | +60 | 25 | 2.4 | 3.0 | +130 | 16 |
| Carbamazepine | 1 | 237.1 | 194.2 | 179.1 | 3.0 | 3.0 | +55 | 25 | 1.2 | 1.0 | +145 | 36/18 |
| Carbamazepine-d_10_* | 1 | 247.1 | 204.2 |  | 3.0 | 3.0 | +55 | 25 | 1.2 | 1.0 | +145 | 36 |
| Chlorpyrifos | 1 | 352.2 | 200.1 | 124.9 | 60.0 | 45.0 | +60 | 27 | 30.0 | 30.0 | +105 | 15/15 |
| Chlorpyrifos-d_10_* | 1 | 362.0 | 201.0 |  | 60.0 | 45.0 | +60 | 27 | 30.0 | 30.0 | +105 | 15 |
| Ciprofloxacin | 1 | 332.1 | 314.1 | 231.1 | 30.0 | 30.0 | +55 | 30 | 9.0 | 12.0 | +135 | 16/36 |
| Ciprofloxacin-d_8_* | 1 | 340.1 | 322.1 |  | 30.0 | 30.0 | +55 | 30 | 9.0 | 12.0 | +135 | 16 |
| Clarithromycin^2^ | 1 | 748.5 | 590.4 | 158.1 | 3.0 | 5.0 | +67 | 40 | 9.0 | 1.0 | +165 | 11/25 |
| Clofibric Acid | 2 | 213.1 | 127.0 |  | 15.0 | 12.0 | -32 | 22 | 6.0 | 12.0 | -82 | 8 |
| Clofibric Acid-d_4_* | 2 | 217.1 | 131.0 |  | 15.0 | 12.0 | -32 | 22 | 6.0 | 12.0 | -82 | 8 |
| Diazinon | 1 | 305.2 | 169.2 | 153.2 | 24.0 | 24.0 | +60 | 30 | 6.0 | 5.0 | +132 | 19/19 |
| Diazinon-d_10_* | 1 | 315.2 | 170.1 |  | 24.0 | 24.0 | +60 | 30 | 6.0 | 5.0 | +132 | 20 |
| 2,4-D | 3 | 219.0 | 161.0 |  | 30.0 | 6.0 | -40 | 20 | 3.0 | 12.0 | -75 | 5 |
| 2,4-D ^13^C_6_* | 3 | 225.0 | 167.0 |  | 30.0 | 6.0 | -40 | 20 | 3.0 | 12.0 | -75 | 5 |
| Diclofenac | 3 | 293.9 | 250.0 |  | 30.0 | 30.0 | -60 | 30 | 9.0 | 12.0 | +85 | 5 |
| Diclofenac-d_4_* | 3 | 298.0 | 254.0 |  | 30.0 | 30.0 | -60 | 30 | 9.0 | 12.0 | +85 | 5 |
| Enrofloxacin | 1 | 360.1 | 342.0 | 316.0 | 30.0 | 30.0 | +80 | 30 | 9.0 | 12.0 | +140 | 18/18 |
| Enrofloxacin-d_5_* | 1 | 365.1 | 347.0 |  | 30.0 | 30.0 | +80 | 30 | 9.0 | 12.0 | +140 | 19 |
| Erythromycin^2^ | 1 | 734.5 | 158.0 |  | 15.0 | 30.0 | +100 | 65 | 3.0 | 9.0 | +155 | 33 |
| Erythromycin-H_2_O^2^ | 1 | 716.5 | 558.4 |  | 15.0 | 30.0 |  |  | 3.0 | 9.0 | +155 | 11 |
| Estradiol | 2 | 271.0 | 145.0 |  | 45.0 | 45.0 | -100 | 60 | 9.0 | 6.0 | -195 | 40 |
| Estradiol-d_4_* | 2 | 275.0 | 147.1 |  | 45.0 | 45.0 | -100 | 60 | 9.0 | 6.0 | -195 | 39 |
| Estrone | 2 | 269.0 | 145.0 |  | 27.0 | 21.0 | -100 | 50 | 6.0 | 4.5 | -180 | 38 |
| Estrone-d_4_* | 2 | 273.1 | 147.1 |  | 27.0 | 21.0 | -100 | 50 | 6.0 | 4.5 | -180 | 36 |
| Ethinylestradiol | 2 | 295.0 | 145.0 |  | 60.0 | 45.0 | -100 | 57 | 6.0 | 9.0 | -176 | 40 |
| Ethinylestradiol-d_4_* | 2 | 299.0 | 147.0 |  | 60.0 | 45.0 | -100 | 57 | 9.0 | 12.0 | -176 | 40 |
| Fenoprofen^1^ | 3 | 241.1 | 197.0 |  | 30.0 | 30.0 | -30 | 15 | 9.0 | 12.0 | -70 | 0 |
| Fluoxetine | 1 | 310.3 | 148.1 |  | 45.0 | 30.0 | +30 | 14 | 18.0 | 15.0 | +92 | 5 |
| Fluoxetine-d_6_*^3^ | 1 | 316.2 | 154.2 |  | 45.0 | 30.0 | +30 | 14 | 18.0 | 15.0 | +92 | 4 |
| Gemfibrozil | 3 | 249.1 | 121.0 |  | 9.0 | 12.0 | -35 | 16 | 2.1 | 12.0 | -90 | 4 |
| Gemfibrozil-d_6_* | 3 | 255.1 | 121.0 |  | 9.0 | 12.0 | -35 | 16 | 2.1 | 12.0 | -90 | 4 |
| Ibuprofen | 3 | 205.0 | 161.0 |  | 30.0 | 30.0 | -25 | 10 | 15.0 | 12.0 | -70 | 2 |
| Ibuprofen-d_3_*^1^ | 3 | 208.0 | 164.0 |  | 30.0 | 30.0 | -25 | 10 | 15.0 | 12.0 | -70 | 2 |
| Imidacloprid^†^ | 1 | 256.2 | 209.0 | 175.2 | 20.0 | 10.0 | +80 | 25 | 9.0 | 9.0 | +95 | 12/17 |
| Indomethacin^†^ | 3 | 356.0 | 312.0 | 297.0 | 30.0 | 30.0 | -36 | 22 | 9.0 | 12.0 | -80 | 2/8 |
| Ivermectin^†^ | 2 | 873.5 | 567.4 |  | 30.0 | 30.0 | -110 | 40 | 9.0 | 12.0 | -170 | 17 |
| Josamycin*^2^ | 3 | 828.0 | 174.3 |  | 30.0 | 30.0 | 80 | 45 | 12.0 | 15.0 | 80 | 35 |
| Ketoprofen | 3 | 253.0 | 209.0 |  | 30.0 | 30.0 | -30 | 10 | 9.0 | 12.0 | -70 | 1 |
| Ketoprofen-d_4_* | 3 | 257.0 | 213.0 |  | 30.0 | 30.0 | -30 | 10 | 9.0 | 12.0 | -70 | 1 |
| Malathion | 1 | 332.0 | 128.0 |  | 45.0 | 30.0 | +65 | 17 | 24.0 | 20.0 | +130 | 14 |
| Malathion-d_6_* | 1 | 338.0 | 128.0 |  | 45.0 | 30.0 | +65 | 17 | 24.0 | 20.0 | +130 | 14 |
| Metoprolol | 1 | 268.2 | 191.1 | 131.2 | 6.0 | 6.0 | +45 | 36 | 2.1 | 5.0 | +133 | 15/17 |
| Metoprolol-d_7_* | 1 | 275.1 | 191.1 |  | 6.0 | 6.0 | +45 | 36 | 2.1 | 5.0 | +133 | 15 |
| Naproxen | 3 | 229.0 | 170.0 | 185.0 | 6.0 | 6.0 | -17 | 22 | 12.0 | 6.0 | -72 | 11/1 |
| Naproxen-d_3_* | 3 | 232.0 | 173.0 |  | 6.0 | 6.0 | -17 | 22 | 12.0 | 6.0 | -72 | 11 |
| Paroxetine^3^ | 1 | 330.2 | 192.2 |  | 30.0 | 30.0 | +90 | 25 | 9.0 | 12.0 | +145 | 16 |
| Propranolol | 1 | 260.1 | 183.1 | 155.1 | 30.0 | 30.0 | +45 | 25 | 9.0 | 12.0 | +130 | 14/23 |
| Propranolol-d_7_* | 1 | 267.2 | 189.1 |  | 30.0 | 30.0 | +45 | 25 | 9.0 | 12.0 | +130 | 14 |
| Roxithromycin^2^ | 1 | 837.5 | 679.5 | 158.0 | 30.0 | 30.0 | +100 | 45 | 9.0 | 12.0 | +180 | 15/30 |
| Spiramycin^2^ | 1 | 875.6 | 318.3 | 174.2 | 30.0 | 30.0 | +100 | 45 | 9.0 | 12.0 | +220 | 22/32 |
| Sulfachloropyridazine^4^ | 1 | 285.1 | 156.1 | 108.2 | 30.0 | 30.0 | +60 | 22 | 9.0 | 12.0 | +105 | 10/20 |
| Sulfadimethoxine | 1 | 311.1 | 156.0 | 245.0 | 10.0 | 5.0 | +60 | 25 | 9.0 | 5.0 | +125 | 17/15 |
| Sulfadimethoxine-d_6_* | 1 | 317.1 | 162.1 |  | 10.0 | 5.0 | +60 | 25 | 9.0 | 5.0 | +125 | 17 |
| Sulfamethazine | 1 | 279.1 | 186.1 | 156.1 | 6.0 | 3.0 | +65 | 20 | 1.8 | 1.0 | +120 | 13/13 |
| Sulfamethazine-^13^C_6_*^4^ | 1 | 285.1 | 186.1 |  | 6.0 | 3.0 | +65 | 20 | 1.8 | 1.0 | +120 | 13 |
| Sulfamethoxazole | 1 | 254.0 | 156.1 | 108.1 | 6.0 | 3.0 | +65 | 20 | 2.1 | 3.0 | +110 | 11/22 |
| Sulfamethoxazole-d_4_*^5^ | 1 | 258.0 | 160.1 |  | 6.0 | 3.0 | +65 | 20 | 2.1 | 3.0 | +110 | 12 |
| Sulfapyridine | 1 | 250.1 | 156.1 | 145.2 | 2.0 | 3.0 | +60 | 20 | 9.0 | 3.0 | +110 | 13/13 |
| Sulfapyridine-d_4_* | 1 | 254.1 | 160.1 |  | 2.0 | 3.0 | +60 | 20 | 9.0 | 3.0 | +110 | 13 |
| Sulfisoxazole^5^ | 1 | 268.1 | 156.1 | 113.1 | 30.0 | 30.0 | +70 | 20 | 9.0 | 12.0 | +105 | 8/12 |
| Triclosan | 2 | 286.9 | 35.0 |  | 30.0 | 30.0 | -55 | 25 | 9.0 | 12.0 | -72 | 9 |
| Triclosan-^13^C_12_* | 2 | 298.9 | 35.0 |  | 30.0 | 30.0 | -55 | 25 | 9.0 | 12.0 | -72 | 9 |
| Trimethoprim | 1 | 291.1 | 230.1 | 261.1 | 9.0 | 6.0 | +80 | 30 | 2.7 | 2.0 | +150 | 21/20 |
| Trimethoprim-d_3_* | 1 | 294.1 | 230.1 |  | 9.0 | 6.0 | +80 | 30 | 2.7 | 2.0 | +150 | 21 |
| Tylosin^2^ | 1 | 916.0 | 772.5 | 174.1 | 60.0 | 45.0 | +110 | 52 | 30.0 | 30.0 | +220 | 28/34 |

LOQ = Limit of quantification

Table S2: Mean concentrations of pharmaceuticals measured by SPE and POCIS in 2012 at sites in the Grand Marais wetland treatment area. Only those compounds that were quantifiable are shown in the table and values are in ng/L (± SD); NA = not available or not sampled, ND = non-detect, <LOQ = below the limit of quantitation^a^.

| **Compound** | **Sampling date** | **Sample**  **type** | **Site** | | | | | | | | | |
| --- | --- | --- | --- | --- | --- | --- | --- | --- | --- | --- | --- | --- |
|  |  |  | **Lagoon** | | **Release** | | **Mid**-**Channel** | **Channel** | **East Wetland** | | **West Wetland** | **Outlet** |
| **2, 4 - D** | May 22/12 | SPE | 7.8 ± 1.0 | | NA | | NA | NA | NA | | NA | ND |
|  | June 15/12 | POCIS | <LOQ | | NA | | NA | NA | NA | | NA | ND |
|  | June 15/12 | SPE | 13 ± 0.4 | | NA | | NA | NA | NA | | NA | <LOQ |
|  | July 16/12 | SPE | NA | | 8.3 ± 1 | | 8.6 ± 1 | NA | 9.3 ± 0.6 | | 7.4 ± 2 | <LOQ |
|  | July 23/12 | SPE | NA | | 7.6 ± 1 | | <LOQ | <LOQ | <LOQ | | <LOQ | ND |
|  | July 25/12 | POCIS | NA | | <LOQ | | NA | ND | ND | | ND | ND |
|  | Aug. 1/12 | SPE | NA | | 5.1 ± 1 | | <LOQ | ND | <LOQ | | <LOQ | ND |
| **Atrazine** | May 22/12 | SPE | 7.3 ± 0.1 | | NA | | NA | NA | NA | | NA | 5.1 ±0.1 |
|  | June 15/12 | POCIS | 3.7 ± 0.03 | | NA | | NA | NA | NA | | NA | 5.4 ± 0.1 |
|  | June 15/12 | SPE | 15 ± 0.1 | | NA | | NA | NA | NA | | NA | 3.1 ± 0.1 |
|  | July 16/12 | SPE | NA | | 6.2 ± 0.3 | | 10 ± 1 | NA | 4.7 ± 0.1 | | <LOQ | <LOQ |
|  | July 23/12 | SPE | NA | | 6.6 ± 0.5 | | 5.6 ± 0.1 | 6.2 ± 0.1 | 6.9 ± 0.2 | | 4.0 ± 0.1 | <LOQ |
|  | July 25/12 | POCIS | NA | | 4.8 ± 2 | | NA | <LOQ | 2.6 ± 1 | | 6.6 ± 2 | <LOQ |
|  | Aug. 1/12 | SPE | NA | | 4.9 ± 0.4 | | 3.5 ± 0.4 | 2.4 ± 0.03 | 2.2 ± 0.2 | | 3.1 ± 0.1 | <LOQ |
| **Carbamazepine** | May 22/12 | SPE | 3.8×10^2^ ± 17 | | NA | | NA | NA | NA | | NA | ND |
|  | June 15/12 | POCIS | 59 ± 20 | | NA | | NA | NA | NA | | NA | ND |
|  | June 15/12 | SPE | 2.1×10^2^± 0.9 | | NA | | NA | NA | NA | | NA | 3.1 ± 0.1 |
|  | July 16/12 | SPE | NA | | 86 ± 2 | | 65 ± 6 | NA | 62 ± 1 | | 8 ± 1 | 4.1 ± 0.2 |
|  | July 23/12 | SPE | NA | | 85 ± 3 | | 72 ± 1 | 85 ± 2 | 12 ± 0.3 | | 58 ± 1 | <LOQ |
|  | July 25/12 | POCIS | NA | | 5.0×10^2^ ±165 | | NA | 49 ± 7 | 49 ± 13 | | 22 ± 0.5 | 22 ± 10 |
|  | Aug. 1/12 | SPE | NA | | 90 ± 2 | | 83 ± 3 | 51 | 49 ± 2 | | 11 ± 0.4 | <LOQ |
| **Gemfibrozil** | May 22/12 | SPE | 1.4×10^2^ ± 10 | NA | | NA | | NA | | NA | NA | ND |
|  | June 15/12 | POCIS | 34 ± 3 | NA | | NA | | NA | | NA | NA | ND |
|  | June 15/12 | SPE | 37 ± 0.8 | NA | | NA | | NA | | NA | NA | ND |
|  | July 16/12 | SPE | NA | 15 ± 2 | | 11 ± 0.7 | | NA | | 12 ± 0.5 | ND | ND |
|  | July 23/12 | SPE | NA | 13 ± 0.4 | | 12 ± 0.7 | | 13 ± 0.6 | | ND | 6.6 ± 0.2 | ND |
|  | July 25/12 | POCIS | NA | 19 ± 6 | | NA | | <LOQ | | <LOQ | ND | ND |
|  | Aug. 1/12 | SPE | NA | 15 ± 0.7 | | 14 ±1 | | 10 ± 0.1 | | 4.1 ± 0.4 | 3.4 ± 0.3 | <LOQ |
| **Sulfamethoxazole** | May 22/12 | SPE | 15 ± 2 | NA | | NA | | NA | | NA | NA | ND |
|  | June 15/12 | POCIS | ND | NA | | NA | | NA | | NA | NA | ND |
|  | June 15/12 | SPE | 12 ± 4 | NA | | NA | | NA | | NA | NA | ND |
|  | July 16/12 | SPE | NA | 21 ± 4 | | 14 ± 3 | | NA | | <LOQ | <LOQ | <LOQ |
|  | July 23/12 | SPE | NA | 10 ± 2 | | 58 ± 6 | | 12 ± 1 | | ND | ND | ND |
|  | July 25/12 | POCIS | NA | ND | | NA | | ND | | ND | ND | ND |
|  | Aug. 1/12 | SPE | NA | 17 ± 7 | | <LOQ | | <LOQ | | <LOQ | <LOQ | <LOQ |
| **Sulfapyridine** | May 22/12 | SPE | <LOQ | NA | | NA | | NA | | NA | NA | ND |
|  | June 15/12 | POCIS | ND | NA | | NA | | NA | | NA | NA | 7.9 ± 5 |
|  | June 15/12 | SPE | <LOQ | NA | | NA | | NA | | NA | NA | ND |
|  | July 16/12 | SPE | NA | <LOQ | | ND | | NA | | <LOQ | ND | ND |
|  | July 23/12 | SPE | NA | ND | | <LOQ | | ND | | ND | ND | ND |
|  | July 25/12 | POCIS | NA | ND | | NA | | ND | | ND | ND | ND |
|  | Aug. 1/12 | SPE | NA | ND | | ND | | ND | | ND | ND | ND |

^a^ LOQs provided in Supplementary Information from Carlson *et al.,* 2013 [4].

Table S3: Primers used for qPCR analysis of ARGs in samples collected in 2012 from the Grand Marais treatment wetland study area.

| Primer | Forward Sequence | Reverse Sequence | Annealing  Temp. (°C) | Reference |
| --- | --- | --- | --- | --- |
| *sul-*I | cgcaccggaaacatcgctgcac | tgaagttccgccgcaaggctcg | 65.0 | Pei *et al.,* 2006 |
| *sul-*II | tccggtggaggccggtatctgg | cgggaatgccatctgccttgag | 57.5 | Pei *et al.,* 2006 |
| *sul-*III | tccgttcagcgaattggtgcag | ttcgttcacgccttacaccagc | 61.0 | Pei *et al.,* 2006 |
| *tet*-M | Mixture of primers for detecting: | *tet*(B), *-*(C), *-*(D), all efflux pumps | 60.0 | Ng *et al.,* 2001 |
| *tet*-O | Mixture of primers for detecting: | *tet*(A), -(E), -(G), all efflux pumps | 60.0 | Ng *et al.,* 2001 |
| *tet*-Q | Mixture of primers for detecting: | *tet*(K), -(L), efflux pumps;  *tet*(M), -(O), -(S), ribosomal protection proteins | 60.0 | Ng *et al.,* 2001 |
| *tet*-W | Mixture of primers for detecting: | *tet*A(P), -(Q), ribosomal protection proteins; *tet*(X), enzyme | 60.0 | Ng *et al.,* 2001 |
| *bla*_CTX_ | ATGTGCAGTACCAGTAATGTKATGGC | ATCACKCGGTTCGCCNGGTAT | 72.0 | Knapp *et al.,* 2010 |
| *bla*_SHV_ | TTGATTTATCTGCGGGATACG | GGAATAAGGGCGACA | 76.0 | Knapp *et al.*, 2010 |
| *bla*_TEM_ | TCGGGGAAATGTGCG | GGAATAAGGGCGACA | 72.0 | Knapp *et al.*, 2010 |
| 16S-rRNA | ACTCCTACGGGAGGGCAG | GACTACCAGGGTATCTAATCC | 60.0 | Knapp *et al.*, 2010 |

Table S4: Abundance of bacterial 16S-rRNA genes and proportion of resistance genes per 16S-rRNA (log (# of genes per mL of water)) in samples collected from the Grand Marais treatment system in 2012. Standard deviations are presented in brackets.

| **Site** | **Date** | **16S ^a^** | ***bl*a_CTX_** | ***bla_SHV_*** | ***sul*-I** | ***sul*-II** | ***sul*-III** | ***bla_TEM_*** | ***tet-*M** | ***tet-*O** | ***tet-*Q** | ***tet-*W** |
| --- | --- | --- | --- | --- | --- | --- | --- | --- | --- | --- | --- | --- |
| **Lagoon** | June 16 | 6.58 (±0.34) | -5.10 (±0.34) | -2.50 (±0.20) | -2.75 (±0.24) | -2.25 (±0.24) | -3.78 (±0.23) | -2.07 (±0.05) | -1.67 (±0.04) | -2.05  (±0.44) | -3.99  (±0.41) | -2.90  (±0.31) |
| **Release** | July 16 | 6.69 (±0.10) | -5.37 (±0.39) | -3.40 (±0.12) | -2.89 (0.15) | -2.39 (±0.15) | -4.80 (±0.56) | -3.09 (±0.37) | -2.33 (±0.10) | -2.44 (±0.44) | -4.33 (±0.34) | -3.58 (±0.17) |
|  | July 23 | 6.08 (±0.64) | -4.95 (±0.62) | -2.93 (±0.69) | 3.12 (±0.09) | 2.62 (±0.09) | -5.74 (±0.46) | -2.15 (±0.63) | -2.34 (±0.39) | -2.55 (±0.63) | -4.33 (±0.20) | -2.35 (±0.47) |
|  | Aug 1 | 6.19 (±0.08) | -5.12 (±1.10) | -4.03 (±0.26) | -3.09 (±0.08) | -2.59 (±0.08) | -5.04 (±0.59) | -2.60 (±0.11) | -2.53 (±0.30) | -2.19 (±0.09) | -4.36 (±0.95) | N/A |
| **Mid -Channel** | July 23 | 6.80 (±0.06) | -5.38 (±0.52) | -3.28 (±0.38) | -3.17 (0.04) | -2.67 (±0.04) | -4.21 (±0.48) | -2.64 (±0.05) | -2.23 (±0.14) | -2.10 (±0.29) | -4.29 (±0.49) | -3.20 (±0.46) |
|  | Aug 1 | 6.83 (±0.09) | -5.63 (±0.13) | -3.09 (±0.08) | -4.45 (±1.67) | -3.95 (±1.67) | -4.31 (±0.36) | -2.79 (±0.27) | -2.54 (±0.09) | -2.56 (±0.27) | -4.89 (±0.71) | -3.16 (±0.16) |
| **Channel** | July 16 | 6.70 (±0.14) | -5.90 (±0.10) | -3.38 (±0.18) | -2.84 (±0.12) | -2.34 (±0.12) | -5.88 (±0.40) | -2.56 (±0.07) | -2.47 (±0.32) | -3.17 (±0.63) | -4.56 (±0.24) | -3.04 (±0.53) |
|  | July 23 | 6.75 (±0.07) | 5.94 (±0.30) | -3.79 (±0.26) | -3.20 (±0.08) | -2.70 (±0.08) | -4.95 (±0.60 | -3.11 (±0.23) | -2.49 (±0.22) | -2.86 (±0.53) | -4.36 (±0.15) | -2.83 (±0.21) |
|  | Aug 1 | 6.54 (±0.28) | -5.80 (±0.31) | -4.10 (±0.12) | -4.09 (±2.05) | 3.59 (±2.05) | -4.73 (±0.69) | -2.69 (±0.33) | -2.63 (±0.11) | -2.42 (±0.52) | -4.33 (±0.53) | N/A |
| **East Wetland** | July 16 | 6.88 (±0.15) | -5.72 (±0.08) | -3.23 (±0.40) | -3.36 (±0.16) | -2.86 (±0.16) | -5.65 (±0.56) | -2.63 (±0.21) | -2.25 (±0.32) | -2.45 (±0.09) | -4.61 (±0.17) | -2.66 (±0.17) |
|  | July 23 | 6.69 (±0.34) | -5.29 (±0.43) | -3.05 (±0.71) | -2.94 (±0.33) | -2.44 (±0.33) | -4.96 (±1.01) | -2.35 (±0.27) | -2.42 (±0.37) | -2.47 (±0.34) | -4.39 (±0.24) | -3.28 (±0.34) |
|  | Aug 1 | 6.58 (±0.18) | -5.35 (±0.26) | -3.71 (±0.47) | -3.91 (±1.74) | -3.41 (±1.74) | -5.83 (±0.73) | -2.57 (±0.13) | -2.10 (±0.21) | -2.22 (±0.19) | -5.04 (±0.88) | -3.32 (±0.37) |
| **West Wetland** | July 16 | 6.77 (±0.03) | -5.06 (±0.49) | -2.79 (±0.04) | -2.88 (±0.03) | -2.38 (±0.03) | -4.21 (±0.07) | -2.67 (±0.02) | -2.25 (±0.14) | -2.01 (±0.13) | -4.18 (±0.09) | -3.34 (±0.10) |
|  | July 23 | 6.64 (±0.14) | -5.08 (±0.32) | -2.95 (±0.55) | -2.75 (±0.19) | -2.25 (±0.19) | -4.46 (±0.32) | -2.50 (±0.27) | -1.92 (±0.19) | -2.08 (±0.18) | -4.14 (±0.04) | -3.02 (±0.12) |
|  | Aug 1 | 6.78 (±0.22) | -5.65 (±0.19) | -3.35 (±0.31) | -3.27 (±0.06) | -2.77 (±0.06) | -4.65 (±0.11) | -2.66 (±0.17) | -5.46 (±0.06) | -2.06 (±0.25) | -4.76 (±0.34) | -2.91 (±0.16) |
| **Outlet** | June 15 | 5.59 (± 0.21) | -3.95 (±0.17) | N/A | -2.85 (±0.15) | -2.35 (±0.15) | -4.16 (±0.58) | -2.27 (±0.26) | -1.91 (±0.13) | -1.93 (±0.26) | -3.83 (±0.43) | -3.19 (±0.33) |
|  | July 16 | 5.84 (±0.38) | -4.92 (±N/A) | -3.76 (±0.62) | -2.89 (±0.43) | -2.39 (±0.43) | -4.12 (±0.45) | -2.13 (±0.29) | -2.73 (±1.22) | -2.39 (±1.02) | -3.51 (±0.55) | -2.50 (±0.20) |
|  | July 23 | 6.16 (±0.17) | -4.87 (±0.32) | -4.06 (±0.21) | -3.04 (±0.24) | -2.54 (±0.24) | -4.29 (±0.25) | -2.66 (±0.16) | -2.33 (±0.21) | -2.40 (±0.18) | -3.73 (±0.13) | -2.86 (±0.14) |
|  | Aug 1 | 6.13 (±0.16) | -4.80 (±N/A) | -4.30 (±0.30) | -1.39 (±4.72) | -0.89 (±4.72) | -4.43 (±0.29) | -2.44 (±0.09) | -2.18 (±0.26) | -2.61 (±0.77) | -4.03 (±0.21) | -2.94 (±0.24) |

^a^ Abundance of 16S-rRNA presented as genes per mL of water.
